# Supplementary material for: Plasmonic Metamaterial Ag Nanostructures on a Mirror for Colorimetric Sensing
Source: Nanomaterials (Basel). 2023 May 16;13(10):1650. doi: 10.3390/nano13101650 (PMC10220596; doi:10.3390/nano13101650)
Supplement: Supplementary file 1 [file nanomaterials-13-01650-s001.zip › nanomaterials-2332233-supplementary.pdf]

Supplementary Materials

Plasmonic Metamaterial Ag Nanostructures on a Mirror for Colorimetric Sensing

Sayako Maeda, Noboru Osaka, Rei Niguma, Tetsuya Matsuyama, Kenji Wada, and Koichi Okamoto* *Correspondence: OkamoT@omu.ac.jp; Tel.: +81-72-254-9263

Department of Physics and Electronics, Osaka Metropolitan University, Osaka 599-8531, Japan

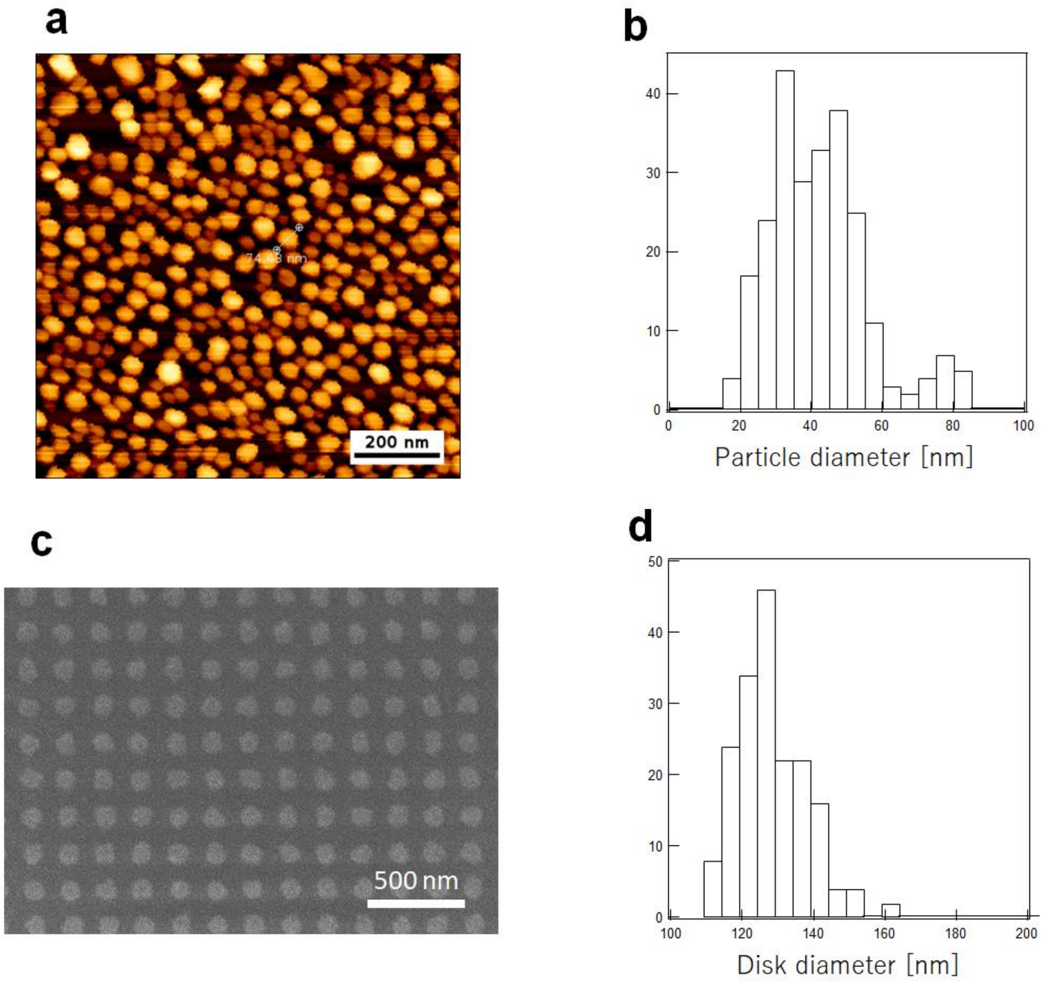

Figure S1. (a) AFM images and (b) histograms of diameters of the nano-hemispheres in NHoM structures shown in Figure 5d. (c) SEM images and (d) histograms of diameters of the nano-disks in NDoG structures shown in Figure 5e.
